# Ecological implications of the pink salmon invasion in northern Norway—Aggregative responses and terrestrial transfer by white‐tailed eagles

**DOI:** 10.1002/ece3.70001

**Published:** 2024-07-21

**Authors:** Bror Mathias Bonde, Audun Stien

**Affiliations:** ^1^ Department of Arctic and Marine Biology, Faculty of Biosciences, Fisheries and Economics UiT – The Arctic University of Norway Tromsø Norway

**Keywords:** invasion ecology, nutrient transfer, resource subsidies, scavenger community

## Abstract

Over the last 10 years, the spawning population of invasive pink salmon (*Oncorhynchus gorbuscha*) has increased in the river systems in northern Norway to a level that is causing concern about their impact on endemic fauna and ecosystem processes. The scale of transfer of pink salmon carcasses into the terrestrial ecosystem is likely to be a key determinant of terrestrial impact. Bears (*Ursus* sp.) are responsible for most such transfers in North America but are rare in Norway. The white‐tailed eagle (*Haliaeetus albicilla*) is common however, and a candidate to be a main cause of such transfers. To evaluate this hypothesis, data on the abundance of white‐tailed eagles and pink salmon were collected along the river Skallelv in northern Norway in 2021, a year the pink salmon spawned in the river, and in 2022, a year no pink salmon spawned in the river. The abundance of white‐tailed eagles along the river was much higher the year pink salmon spawned in the river. Furthermore, white‐tailed eagles were observed aggregating and catching pink salmon where and when pink salmon were present at the spawning and post‐spawning stages. Based on our observations, we suggest that the white‐tailed eagle is the main species involved in the transport of pink salmon from the river into the riparian zone in northern Norway and that other scavengers, in particular the red fox (*Vulpes vulpes*) and common raven (*Corvus corax*), play an important role in transporting pink salmon carcasses from the riparian zone to the wider terrestrial ecosystem.

## Graphical abstract

The impact of the invasive pink salmon on terrestrial ecosystem processes in northern Norway is largely unknown. We find that the white‐tailed eagle is likely to be the main species involved in the transport of pink salmon from the river into the riparian zone in northern Norway and that other scavengers, in particular the red fox (*Vulpes vulpes*) and common raven (*Corvus corax*), play an important role in transporting pink salmon carcasses from the riparian zone to the wider terrestrial ecosystem.

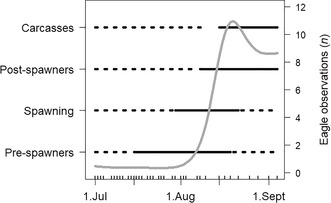

## 1 INTRODUCTION

**FIGURE 1:**
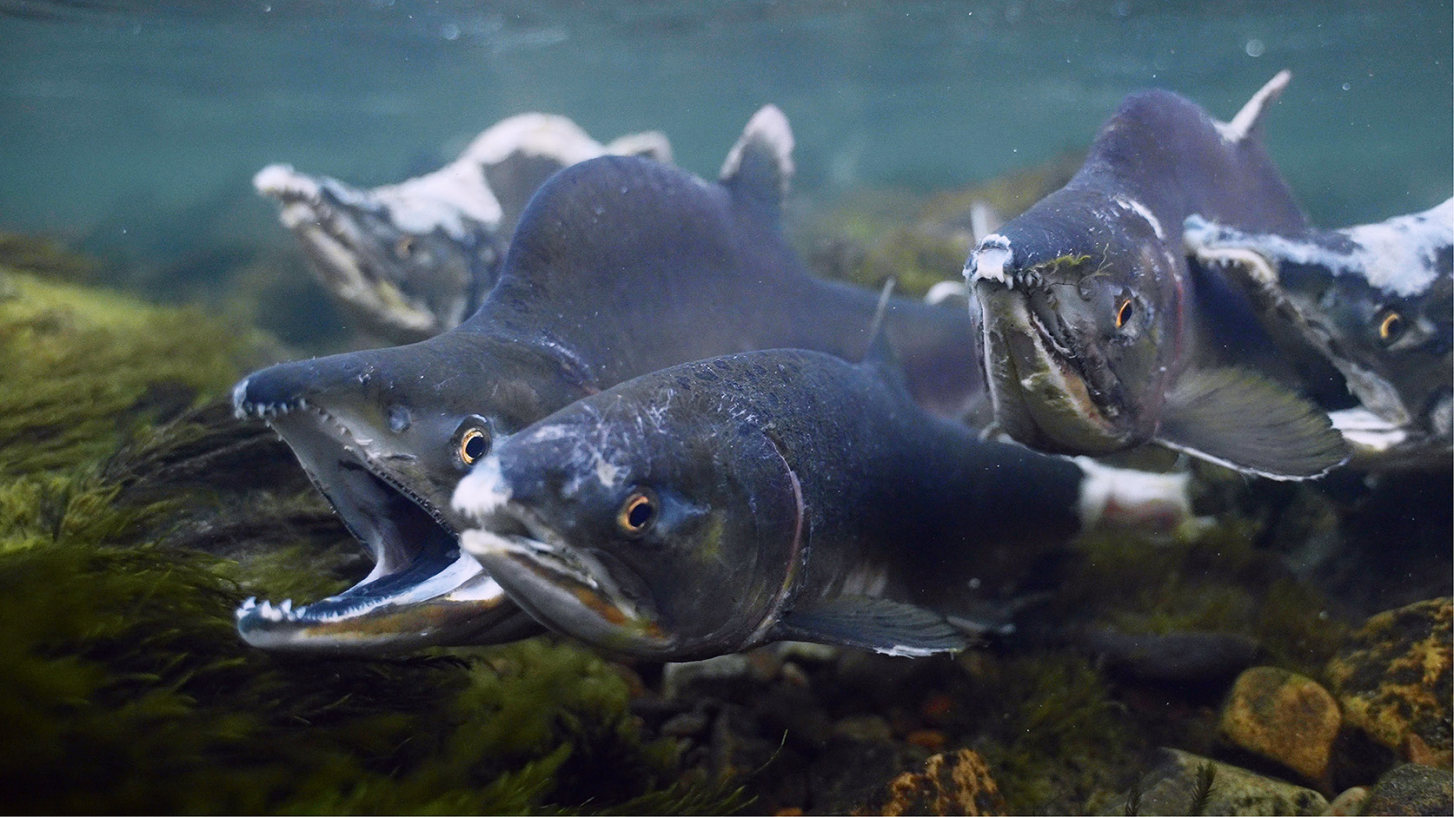
Pink salmon (*Oncorhynchus gorbuscha*) (Photo: Skjalg‐Helmer Vian).

Terrestrial Arctic ecosystems are low‐productive systems with simple trophic structures but are often involved in resource exchanges with more productive neighbouring ecosystems (Gauthier et al., 2011; Giroux et al., 2012; Killengreen et al., 2011). Such cross‐boundary resource subsidies are important for ecosystem structure, function and regulation in terrestrial arctic ecosystems (Gauthier et al., 2011; Killengreen et al., 2012). Seasonal migration is a key process that links terrestrial arctic food webs to more productive surrounding ecosystems (Giroux et al., 2012). The spawning migrations by native anadromous salmonids (*Salmo salar*, *Salmo trutta* and *Salvelinus alpinus*) are, however, regarded to have minor effects on the nutrient dynamics in the neighbouring terrestrial ecosystems in northern Fennoscandia (Jonsson & Jonsson, 2003). In contrast, Pacific salmon (*Oncorhynchus* spp.) are well known for their cross‐boundary transfer of resources to terrestrial ecosystems in North America (Helfield & Naiman, 2006; Quinn et al., 2009; Reimchen, 2017). The recent invasion by pink salmon (*Oncorhynchus gorbuscha*, Figure 1) in Norwegian rivers may therefore result in increased nutrient transfer from marine to terrestrial ecosystems surrounding invaded rivers (Jonsson & Jonsson, 2003).

Pacific salmon (*Oncorhynchus* spp.) are anadromous and semelparous salmonids, whereof the pink salmon is the most numerous (Ruggerone et al., 2023). Pacific salmon species have a substantial impact on their native marine (Ruggerone et al., 2023; Springer et al., 2018), freshwater (Walsh et al., 2020) and adjacent terrestrial ecosystems (Walsh et al., 2020) through their impacts on nutrient flow, both as predators, prey and carrion. The pink salmon has expanded its range from Northern Russia into the North Atlantic Ocean and European waters (Lennox et al., 2023). In particular, in northern Norway, the spawning population of pink salmon has increased dramatically in recent years (Berntsen et al., 2018, 2020, 2022; Diaz Pauli et al., 2023; Sandlund et al., 2019) to a level where it causes concerns with respect to its impact on endemic species and ecosystem integrity (Hindar et al., 2020; Lennox et al., 2023). The impact of invasive pink salmon on Norwegian terrestrial ecosystems has so far received little attention from both the scientific‐ and management communities (Hindar et al., 2020), however, Dunlop et al. (2021) followed pink salmon carcasses that were experimentally deposited on land next to a river in northern Norway, and documented that both the common raven (*Corvus corax*), hooded crow (*Corvus cornix*) and red fox (*Vulpes vulpes*) scavenged on the available carcasses.

**FIGURE 2:**
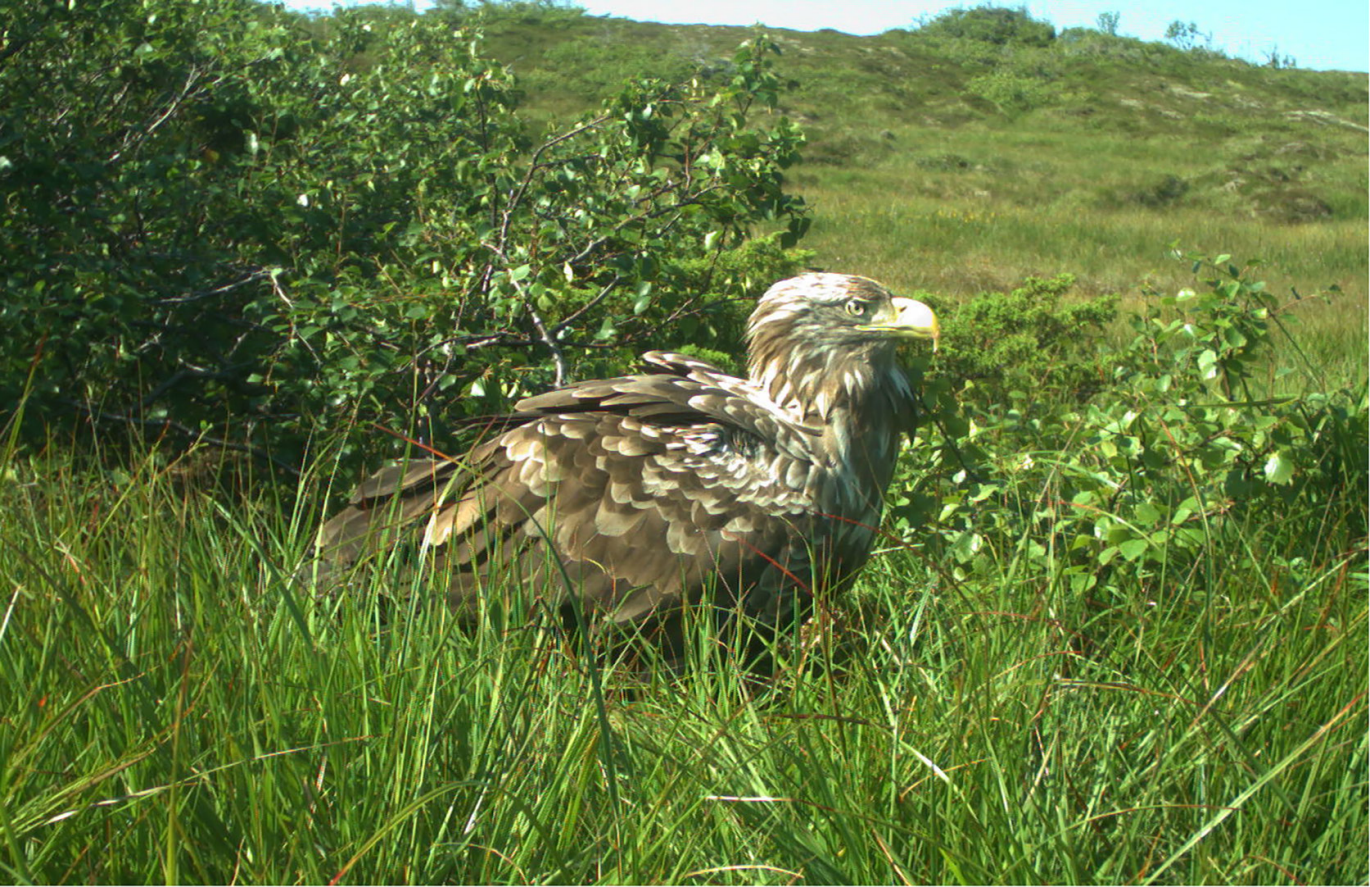
White‐tailed eagle (*Haliaeetus albicilla*).

In North America, brown bears (*Ursus arctos*) and black bears (*Ursus americanus*) are known to be important in the transfer of Pacific salmon (*Oncorhyncus* spp.) from rivers to land, and thereby make the marine‐derived nutrients available to a wide range of terrestrial organisms (Walsh et al., 2020). Bears are rare in Norway and most other European countries that experience, or are likely to experience, pink salmon invasion (Chapron et al., 2014; Dupont et al., 2023). Brown bears are therefore unlikely to become important in the transfer of pink salmon from rivers to land in these areas. Here we suggest that the white‐tailed eagle (*Haliaeetus albicilla*, Figure 2) may be important for such transfer in Norway. Empirical evidence for white‐tailed eagles preying and scavenging on pink salmon in Norwegian river systems is lacking. However, the closely related Bald eagle (*Haliaeetus leucocephalus*), is known to aggregate and feed at Pacific salmon spawning sites in North America (Levi et al., 2015; Restani et al., 2000). Furthermore, the white‐tailed eagle is known to be an efficient scavenger and predator in aquatic environments (Witman et al., 2014), and has previously been suggested to be a scavenger of pink salmon (Dunlop et al., 2021), is abundant in Norway (Folkestad & Probst, 2013; Heggøy & Øien, 2014), and is highly mobile (Duvall, 2022) which allow aggregative responses to abundant food sources (Gutiérrez‐Cánovas et al., 2020).

In this study, we use the biannual presence of spawning runs as a natural case–control design to assess the responses by the terrestrial predator–scavenger community to spawning pink salmon. Observational data were collected in two consecutive years along the river Skallelv in northern Norway, one year with and one without spawning pink salmon in the river. Specifically, we evaluate whether the abundance of white‐tailed eagles along the river differs between the two study years, and within the year with spawning pink salmon present, and whether seasonal changes in white‐tailed eagle abundance are associated with demographic shifts in the pink salmon population. We also report observations of the wider scavenger community along the river and suggest a model for how nutrients from pink salmon enter and spread into the surrounding terrestrial ecosystem.

## 2 MATERIALS AND METHODS

### 2.1 Study area and species

**FIGURE 3:**
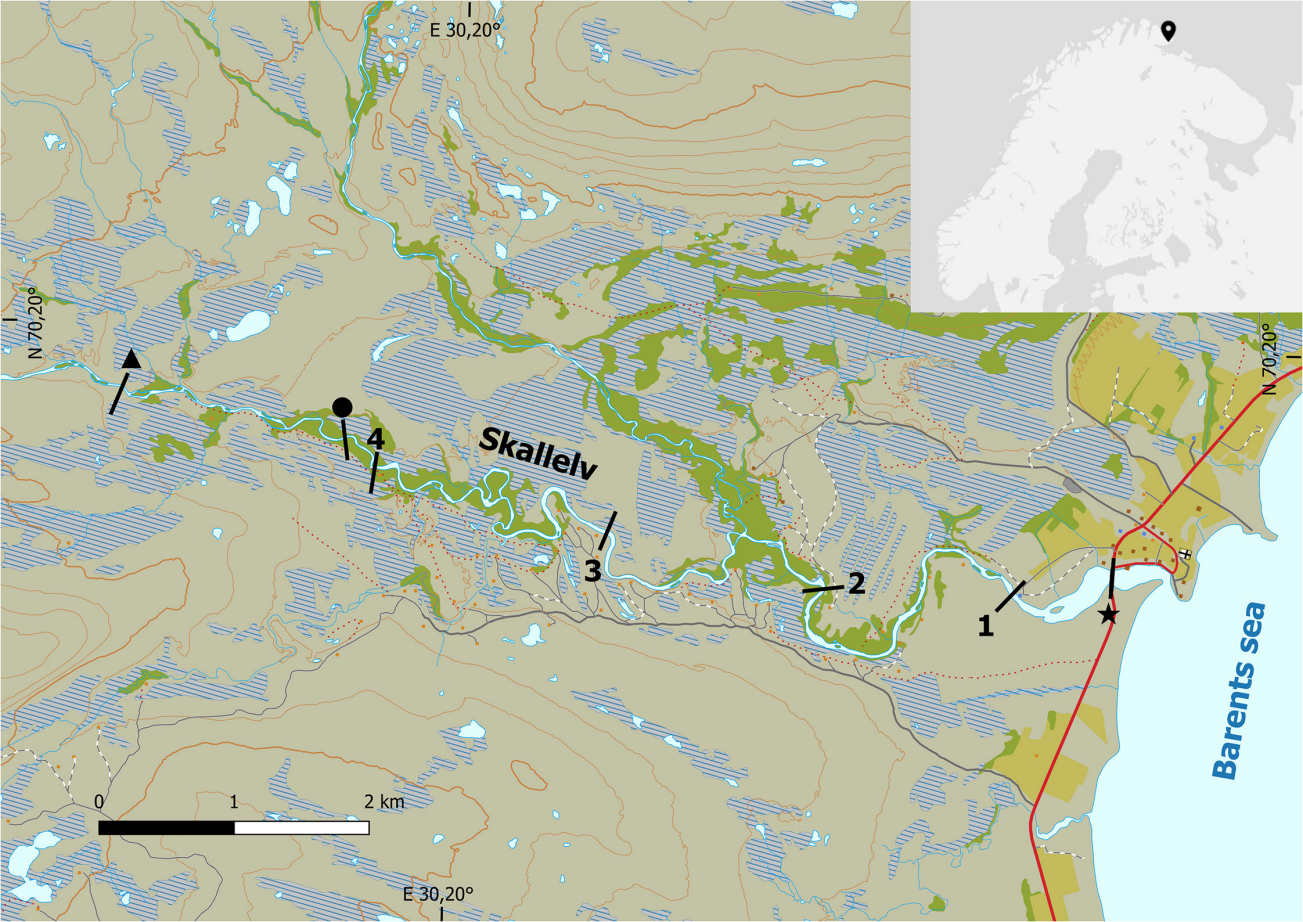
Map of the study area and the geographical location of Skallelv, a sub‐arctic river system on the Eastern coast of the Varanger Peninsula in Norway. The line transect used for white‐tailed eagle counts started at the bridge by the sea (star) and followed the path along the river up to the triangular reference point. The circular reference point marks the top and the bridge (star) marks the low point of the stretch of river covered by drift counts. The numbered sites along the river are river sections referred to in the methods description of the survey of pink salmon demographic stages in the spawning run in 2021.

The study was conducted along Skallelv, a sub‐arctic river system on the east coast of the Varanger Peninsula in Norway (Figure 3). The study area has a rich topography of glacial‐ and marine landforms, mostly characterised by kame terraces, eskers and marine terraces. These features have an influence on the river characteristics of the upper, middle and lower sections of Skallelv. The upper sections are mostly characterised by highly braided riffles with mid‐channel banks, irregular backwater and the confluence of smaller tributaries. The middle section is mostly characterised by meandered channels, with point bars, attached bars and wider channels of shallow, low‐velocity and low‐turbulent water. The lower section is mostly characterised by deeper channels with large pools of slow and low‐turbulent water and with sections of breaks. The study area is dominated by sub‐arctic tundra, although the riparian vegetation has patchy formations of willows (*Salix* sp.) along the lower sections of the main river channel and larger tributaries.

The study area has a set of mammalian terrestrial and semi‐aquatic species as well as avian species that can prey or scavenge on pink salmon (Dunlop et al., 2021). The terrestrial mammal species is the red fox, the semi‐aquatic mammal species are the introduced American mink (*Neovison vison*) and Eurasian otter (*Lutra lutra*), and the avian species are the white‐tailed eagle, common raven and hooded crow.

### 2.2 Study design

Data on white‐tailed eagle and pink salmon abundances and demographic stages were collected in two consecutive seasons using line transect counts and drift counts along Skallelv from July 1st to September 5th in 2021 and 2022. Spawning pink salmon were only present in the river in 2021.

#### 2.2.1 Eagle line transect counts

The line transect surveys followed the footpath along the bank of the Skallelv. The number of observed white‐tailed eagles seen from this footpath was recorded daily from July 1st to September 5th in 2021 and 2022. The transect started at the bridge crossing the estuary at N 70.1862° – E 30.3284° (Figure 3, star) and ended at an upper point at N 70.1967° – E 30.1368° (Figure 3, triangle). The upper point of the transect was set to ensure that the transect covered the distribution of spawning pink salmon in the Skallelv observed in 2019. All eagle line transect counts were completed before noon by the first author. Observations of white‐tailed eagles were included in the count if they were observed: (i) during a predation event in the river or on ground, (ii) during other foraging behaviour, (iii) including scavenging behaviour in the river or on ground, (iv) resting on the ground near the main river channel and (v) in low flight along the main river channel. Observations outside of these criteria were excluded, including: (i) observations of eagles further up the valley from the upper triangle (Figure 3), and (ii) observations of high‐altitude transit flights that crossed the study area.

Data were collected along the line transect 63 days out of 67 from July 1st to September 5th in both 2021 and 2022. Non‐collection of transect data was due to other obligations.

#### 2.2.2 Drift counts

Drift counting has become a standard method for estimating the abundance of salmonids in clear streams and rivers (Orell et al., 2011; Orell & Erkinaro, 2007) and consists of counting salmonids while drifting down the river using snorkelling gear. In Skallelv, drift counts were used to record the abundance, distribution and demography of pink salmon from July 1st to September 5th in 2021 and 2022. All drift counts were made by the first author, who has performed drift counts of salmonids in Skallelv every year since 2018.

The drift count surveys were conducted within a stretch that ranged from an upper point at N 70.1937° – E 30.1792° (Figure 3, circle) to the bridge that crosses the estuary at N 70.1862° – E 30.3284° (Figure 3, star). All drift counts were made in environmental conditions that optimised visibility and were timed to concur with tidal shifts, as most salmon runs occur at high tide (Heard, 1991). In 2021, the total drift length varied in response to the pink salmon population moving upstream over the season. In both July and August, all drift counts included Site 3 (see Figure 3). However, in July the drifts were centred on the areas with high densities of pink salmon from Site 2 (Figure 3) and downstream to the bridge that crosses the estuary. In August, most drifts started at Site 4 (Figure 3). In September, the drifts covered the whole stretch of river as the pink salmon had spread to the whole range and were also observed beyond the upper point of the drift counts (Figure 3). In 2022, all drift counts ran from Site 3, and most covered the whole stretch of river (Figure 3). A total of 37 drift counts were completed over the course of the study period in 2021, and 20 drift counts were made over the course of the study period in 2022.

In 2021, the main objectives of the drift counts were to quantify the number of pink salmon in Skallelv and record seasonal changes in the spatial distribution and demographic composition of the population. The demography of the pink salmon was categorised into five successional stages. (i) The early river stage: bright silver fish not visually distinguishable from individuals at the marine stage, no dorsal hump or curved jaw. (ii) Pre‐spawners: recognised as individuals in a morphological transition phase between the early river and spawning stage; they have lost their bright silver colour and turned a darker colour towards grey or green, males have not yet developed a pronounced dorsal hump or curved jaw (see Figure S1a); females have no marks on their tail‐, anal‐ or pelvic fins from digging into the river bed during nesting activity. (iii) Spawners: dark‐coloured individuals; males have developed a large dorsal hump, are often covered in bite marks, and have a curved jaw with sharp‐elongated teeth (see Figure S1b); females have developed an enlarged abdomen, typically covered in dig marks, and both the tail‐, anal‐ and pelvic fins are worn. In addition, the presence of female spawners is detectable by submerged observers from the sound of their nest‐digging activity. (iv) Post‐spawners; individuals are severely affected by spawning activities and show physical deterioration. Both male and females appear physically diminished, typically having cloudy eyes, open wounds, do not respond with evasive movements when approached during drift counts, and have pronounced deterioration in body areas involved in spawning activities. The entire tail‐, anal‐ and pelvic fins could be missing (see Figure S1e). (v) Carcasses: recognisable by their lifeless state and any movement or position being determined by the current. However, these individuals sometimes drifted and rolled downstream making it more challenging to distinguish post‐spawners and carcasses in these cases.

To count the number of pink salmon in the different stages was methodologically difficult due to very high densities of pink salmon in the river in 2021 (see Figure S1c). It was an upstream shift in the areas with high densities of pink salmon over the study period. All drift counts made after July 10th encountered areas with extreme densities of pink salmon from about 500 metre above Site 1 (Figure 3) and downstream. In drift counts made after July 20th, we also encountered extreme densities from about 500 m below Site 2 and downstream (Figure 3). The drift counts made between July 22nd and August 1st all encountered extreme densities from Site 2 and downstream, and after August 1st pink salmon dispersed further upstream. An approximate count of the spawning population became possible when most of the pink salmon had moved further upstream and dispersed across separate spawning grounds on August 7th. This count suggested that approximately 1000 pink salmon spawned in Skallelv in 2021, while 4494 pink salmon were reported removed from the river by net and rod fishing in 2021. No adult pink salmon were observed during drift counts in 2022.

#### 2.2.3 Observational surveys

When performing the transect line surveys for white‐tailed eagles, observations of other bird and mammalian species were noted. The focus was on species that were likely to prey or scavenge on pink salmon in sub‐arctic river systems. The observations along the transect line included spoor signs such as tracks, faeces and pellets. From mid‐August, 10 sites along the white‐tailed eagle transect were selected for point observations by binoculars (Swarovski CL 10 × 25) on the return trip once the daily line transect had been completed. At these sites, scavengers were recognised when they were observed: (i) foraging on naturally occurring carcasses of pink salmon, (ii) scavenging on pink salmon carcasses left by white‐tailed eagles, (iii) transporting pink salmon carcasses, (iv) posting on bank formations or on levees near the main channel, (v) and when pellets or faeces that contained fish shells and bones were found. In addition, 51 entrances at 13 separate red fox den sites within the study area, were monitored for signs of activity.

### 2.3 Statistical analysis

Count data of white‐tailed eagles from the line transects were summarised by the dates within each season. The seasonal subsets were then plotted to visually inspect the seasonal and annual pattern of white‐tailed eagle counts. The seasonal variation in the data was formally analysed using generalised additive models assuming a Poisson distribution for the eagle counts, a log link function and using a smoothing thin plate spline function to capture nonlinear seasonal changes in average counts, with smoothing parameter selection using REML, as available in the mgcv package (v 1.8‐34; Wood, 2011) in R (v 4.3.2, R Core Team, 2023). The counts were first analysed with year fitted as a categorical predictor variable (factor) and date within the seasons fitted as a numerical predictor variable. The simplest model we fitted included only an additive effect of year. In the more complex model, we included additive non‐linear variation with sample day, modelled using a spline function (*gam(count~year + s(sampleday), family = poisson)*). The most complex model we adopted allowed the non‐linear seasonal pattern in White‐tailed eagle counts to differ between years. (*gam(count~year + s(sampleday, by = year), family = poisson)*). The models were compared using analysis of deviance and assumptions evaluated using residual plots.

For each drift count survey date, we coded a binary variable for each of the four demographic spawning stages (pre‐spawning, spawning, post‐spawning and carcass) that described whether at least one individual at that spawning stage was observed present or not in the drift count. To evaluate whether the seasonal variation in the relative abundance of white‐tailed eagles was related to the demography of pink salmon at Skallelv, we fitted a generalised linear model to the counts of white‐tailed eagles in 2021 and used the four binary spawning stage variables as predictor variables.

## 3 RESULTS

**FIGURE 4:**
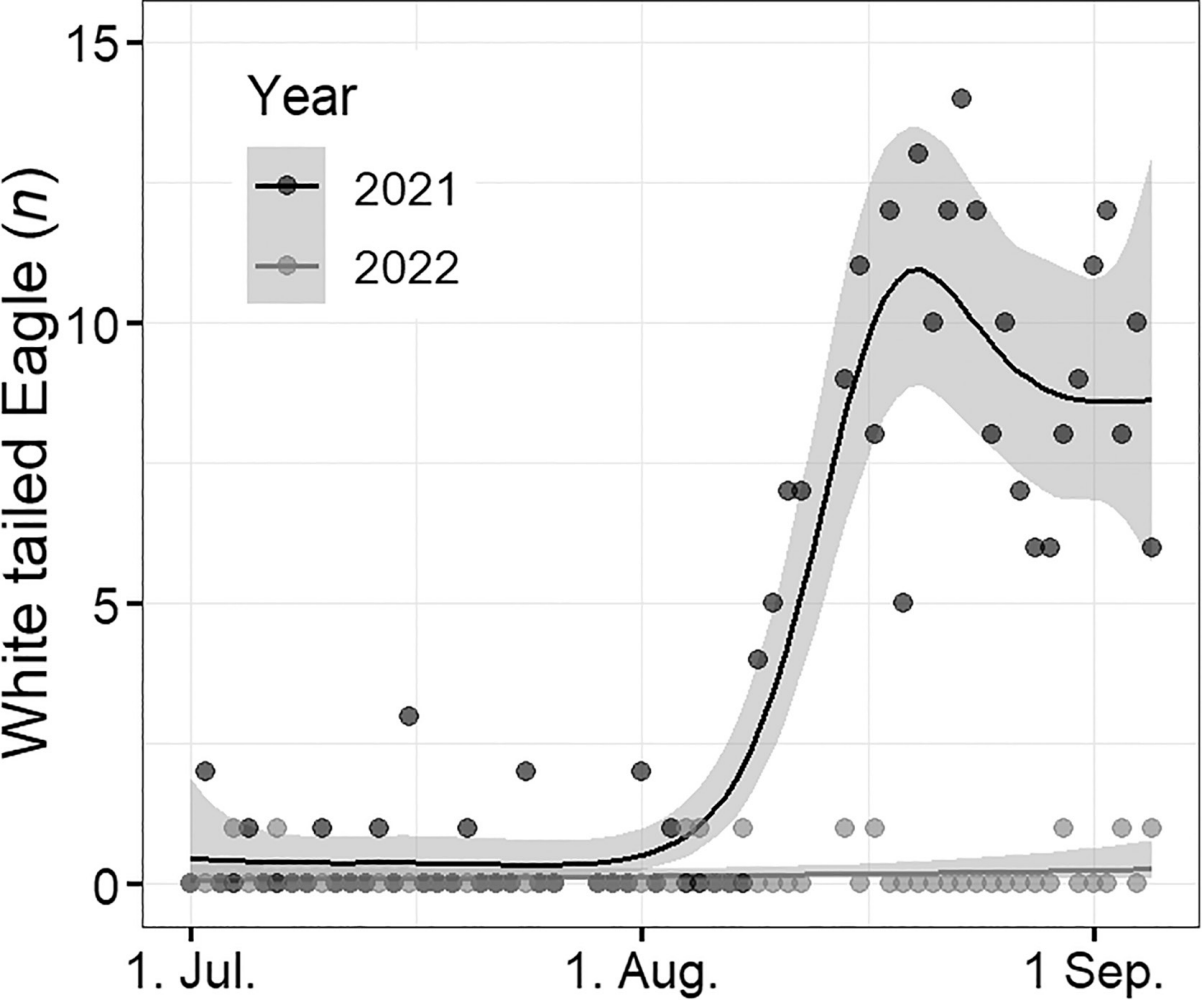
Seasonal variation in the number of observed white‐tailed eagles along the transect line at Skallelv between July 1st and September 5th in 2021 and 2022. The dark grey points and line show the daily observations and regression line for 2021 when spawning pink salmon were present in the river. The lighter grey points and line show the daily observations and regression line for 2022 when spawning pink salmon were not present in the river. Shaded areas show 95 % confidence intervals for estimated mean white‐tailed eagle abundance.

The number of white‐tailed eagles observed along the transect line in Skallelv differed substantially between the 2 years (χ^2^ = 8.7, df = 1.1, *p* < .005). In 2021, when spawning pink salmon were present in the river, the abundance of white‐tailed eagles was initially low in July (mean = 0.38 eagles per day, 95% CI = [0.20, 0.65]), but increased in August to reach a maximum mean of 10.9 eagles observed per day around August 20th (95% CI = [8.9, 13.5]). The numbers remained high in the beginning of September (mean = 9.4 eagles per day, 95% CI = [7.0, 12.3]) until the end of the study period (Figure 4). In contrast, the abundance of white‐tailed eagles was low with no evidence for a seasonal change in abundance in 2022 (mean = 0.16 eagles per day, 95% CI = [0.08, 0.28]), when spawning pink salmon were not present in the river (Figure 4).

**FIGURE 5:**
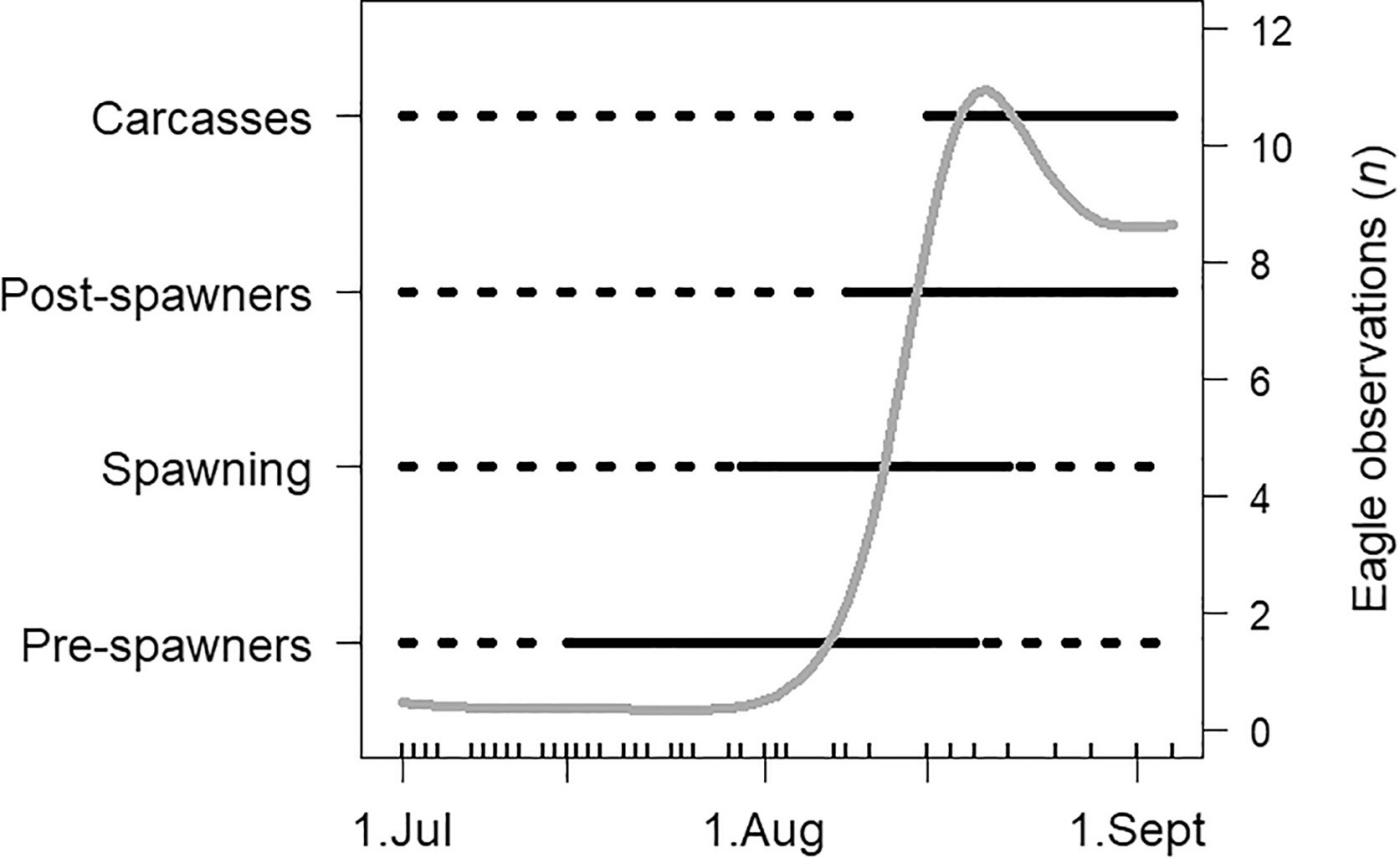
The time periods with observations of the different spawning stages of pink salmon displayed Skallelv in 2021. Solid black lines show periods the stage was present and dotted lines show periods the stage was absent. The estimated regression line for the abundance of white‐tailed eagles (taken from Figure 4) is displayed as a grey line with its scale assigned to the right axis. The dates of the 37 drift counts done are indicated by the inverted marks on the *x*‐axis.

Adult pink salmon in the early river stage were already present in the river at the time of the first drift count on July 1st. Pink salmon at the pre‐spawning stage were first observed on the 15th of July (Figure 5), spawners at the end of July (July 30th), post‐spawners in early August (August 8th) and the first carcasses were sighted in mid‐August (August 15th) (Figure 5). There was substantial overlap in the presence of spawning stages throughout the season (Figure 5).

The observed number of white‐tailed eagles was relatively low when pre‐spawners and spawners were present in the river and increased substantially when post‐spawners and carcasses became present (Table 1, Figure 5). A model for eagle counts was fitted with sample day fitted as a smoothing spline, and the factors describing the presence of post‐spawners and carcasses were included as predictor variables. This model was compared to a model including only the presence of post‐spawners and carcasses as predictors. The comparison suggested that the presence of post‐spawners and carcasses explained most of the seasonal variation in eagle counts, and the estimated smooth term for sample day explaining little additional variation (*p* > .05). This result suggests that a lot of the seasonal variation in white‐tailed eagles can be explained by the presence of post‐spawners and carcasses in the river.

**TABLE 1:** Model estimates from a generalised linear model assuming a Poisson distribution for the daily observations of white‐tailed eagles and a log link function for the relationship between observed numbers of white‐tailed eagles in 2021 and the presence and absence of different pink salmon spawning stages fitted as binary factors.

Parameter	Estimate	Std. error	*p*‐Value
Intercept	−0.62	0.31	
Pre‐spawners	−0.28	0.32	.39
Spawners	0.22	0.32	.48
Post‐spawners	1.58	0.56	.005
Carcasses	1.16	0.49	.02

*Note*: The intercept represents the estimated log (average number of white‐tailed eagles) when only pink salmon at the early river stage was observed in the river.

**FIGURE 6:**
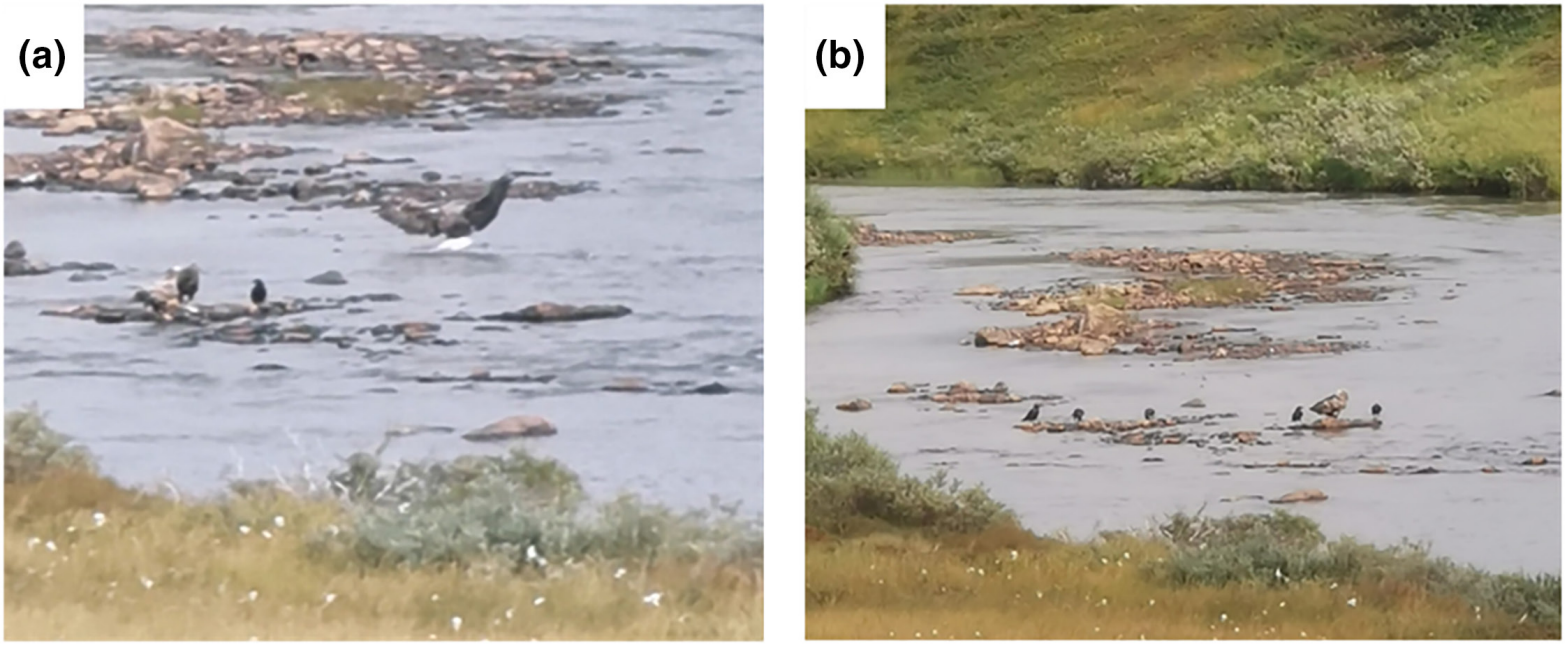
(a) A white‐tailed eagle strikes a pink salmon in an example of a predation event; (b) a lone white‐tailed eagle with five scavenging common ravens at the same site.

During the transect surveys in 2021, white‐tailed eagles were observed to prey upon both spawners and post‐spawners of pink salmon between August 8th and September 5th (Figure 6a). The eagles were observed to wade with their kills ashore, but no observations were made of them transporting the kills far from the riparian zone. Repeatedly used foraging sites had either mid‐channel banks (Figure 6b), point‐ or attached banks that were situated in sections of wider river channels with shallow, low‐velocity and low‐turbulent waterflows.

In addition to white‐tailed eagles, four species were observed to actively scavenge on available pink salmon carcasses from August 8th: the (i) common raven, (ii), hooded crow, (iii) red fox and (iv) American mink. Most of these sightings were made at the foraging sites of white‐tailed eagles where remains of their kills had been left. The common raven was the most abundant and frequently sighted scavenger. Both ravens and hooded crows were regularly observed to transport smaller pieces of pink salmon (e.g. parts of heads, gill lids, jaws, spines) out of the riparian ecosystem. Red foxes were observed to transport considerably larger pieces, including whole carcasses, out of the riparian ecosystem and were observed to cache carcasses at unoccupied den sites near the main river channel. These den sites also became a secondary source of carcasses for the avian scavengers. The American mink was not directly observed to predate on pink salmon, but track signs suggested that predation events occurred. Drag‐marks were found in association with mink tracks that lead to carcasses of post‐spawners of pink salmon with no signs of predation by white‐tailed eagles. The Eurasian otter seemed less important for the cross‐ecosystem transfer of pink salmon in the study system as it was only observed once over the course of the study period in 2021 and no track signs were seen.

No adult pink salmon were observed during drift counts in 2022, but shoals of pink salmon fry were encountered on all drift counts made in July. The number of shoals appeared to decrease over the course of July, with only a few sighted on the last drift count on July 26th. There was no pre‐planned methodology for monitoring the impact of pink salmon fry on the food web. The impression was, however, that the abundance of Arctic terns (*Sterna paradisaea*) was higher along the river in 2022 than in previous years, and that Arctic terns seemed to forage higher upstream than in previous years. In addition, in 2022 the American mink was also observed to actively hunt on smaller fish prey that were most likely pink salmon fry.

## 4 DISCUSSION

**FIGURE 7:**
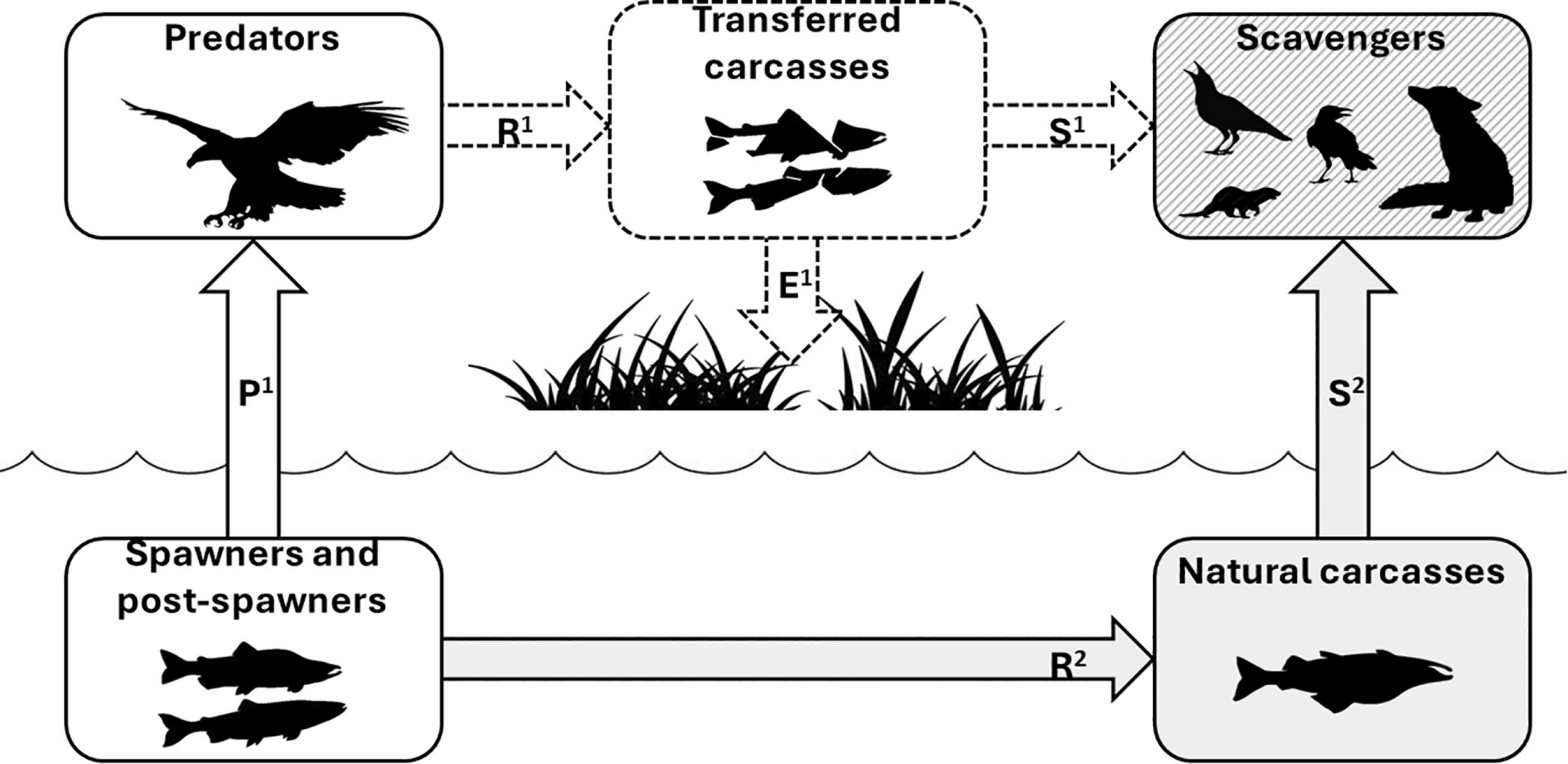
Model for the cross‐boundary transfers of marine‐derived energy, nutrients and organic matter from pink salmon spawning in rivers in northern Norway. The natural conversion of marine‐derived resources is represented by solid arrows, while carcass‐derived resources made available by white‐tailed eagles have dotted arrows; P1 represents predation on spawners and post‐spawners by white‐tailed eagles and R1 describes the terrestrial transport of carcasses by these eagles; E1 represents the nutrient contribution of these carcasses to local plants and soil fauna while S1 represents the scavenging on these carcasses by vertebrates, including the common raven, hooded crows, red fox, otter and American mink; R2 represents the natural production of carcasses from spawned adults and S2 the scavenging of these carcasses by vertebrates.

We found that the abundance of white‐tailed eagles was much higher in Skallelv in late August and early September 2021, when pink salmon spawned in the river, than in 2022, when no spawning occurred. The elevated abundance of white‐tailed eagles was positively related to the presence of pink salmon in spawning and post‐spawning stages. Furthermore, our observations suggest that the white‐tailed eagle was the main species that actively transported pink salmon between the riverine‐ and terrestrial ecosystem and thereby improved the food supply for the wider scavenger community by making carcasses available at their repeatedly used foraging sites (Figure 7). The other scavengers in the system, in particular the common raven and red fox, were more important for the transport of the carcasses out of the riparian zone and into the wider terrestrial sub‐arctic ecosystem.

Large spawning runs of pink salmon are relatively new to northern Norway (Berntsen et al., 2018, 2020, 2022), yet white‐tailed eagles have clearly recognised the spawning runs as a new food source. Visual observations suggested that spawners and post‐spawners were the principal prey items for white‐tailed eagles, but also carcasses were consumed. White‐tailed eagles aggregated along the stretches of river with pink salmon spawning aggregations. In these areas, the white‐tailed eagles were observed to kill pink salmon and wade with their kill ashore. It is likely that the predation by white‐tailed eagles was restricted by the physical characteristics of the stream (e.g. Huxel & Polis, 2013) and that the habitat choice and behaviour of spawning pink salmon make the species vulnerable to white‐tailed eagle predation during and after spawning. In particular, the slow behavioural response of post‐spawners to disturbance probably makes pink salmon easy prey for white‐tailed eagles. Pink salmon generally spawn in water of 30–100 cm depth, but can spawn at 10–15 cm depth (Heard, 1991). In Skallelv, spawners and post‐spawners stayed at spawning sites characterised by shallow water (<50 cm) in wide channels with relatively low‐velocity and low‐turbulent water flows. In contrast, at the early river and pre‐spawning stages they occupied deeper water with reaches of high‐velocity and high‐turbulence water flows. The shallow spawning grounds are likely to facilitate predation by white‐tailed eagles, as it makes it easier for them to detect and catch the salmon. We may therefore hypothesise that white‐tailed eagles are more important for the transfer of nutrients to neighbouring terrestrial ecosystems along smaller rivers with shallow pink salmon spawning grounds, as seen in Skallelv, while white‐tailed eagles may be a less efficient predator along larger rivers. We also note that the abundance of white‐tailed eagles is high in most of Norway, but less common in most other European waters that are likely to be invaded by pink salmon (Folkestad & Probst, 2013). The importance of white‐tailed eagles for the transfer of pink salmon nutrients to neighbouring terrestrial ecosystems is likely to be limited by white‐tailed eagle population in many countries.

The elevated aggregation of white‐tailed eagles along the river coincided in time with the presence of post‐spawning salmon. This may suggest that the presence of post‐spawners was the main attractant for white‐tailed eagles, although both spawners and post‐spawners were observed killed by eagles. Spawners may have been bycatch for eagles attracted to post‐spawners. Alternatively, the eagles may have shown a slow aggregative response to spawners as well as post‐spawners, the main clue being the presence of pink salmon on the shallow spawning grounds, that is, the temporal association with post‐spawners was the result of a delayed response to spawners. We note that the white‐tailed eagle differs from the Bald eagle in North America which feeds on Pacific salmon carcasses (Levi et al., 2015; Restani et al., 2000). However, a third factor that may have affected the timing of white‐tailed eagle aggregation is that the recreational fishing season for resident salmonoids closed on August 8 2021. This resulted in a reduced human‐presence along the river in the latter half of August and could therefore impede our perspective on what caused the rapid shift in the abundance of white‐tailed eagles in 2021.

The white‐tailed eagles generated patches with marine‐derived resources at the repeatedly used foraging sites along the stream, much like the patches generated by Alaskan grizzly bears (*Ursus arctos horribilis*) forging on Pacific salmon (Gende et al., 2004). The scavenger species recorded for pink salmon in northern Norway by Dunlop et al. (2021) were the same species as recorded in our study. A main difference between studies was that pink salmon carcasses were made available to the scavengers by humans in the study design of Dunlop et al. (2021), while white‐tailed eagles made them available to the wider scavenger community in our study. The aggregated spatiotemporal distribution of carcasses produced by the white‐tailed eagles can be expected to cause local effects, both through the attraction of other scavengers and by generating biogeochemical hotspots affecting the productivity and biodiversity of the soil ecosystem, including plant growth (Johnson‐Bice et al., 2023).

The pink salmon represent a new subsidy to the terrestrial ecosystem in northern Norway and the spawning population of pink salmon in Norway is projected to continue to increase (Diaz Pauli et al., 2023). The result of our study is consistent with the previous findings by Dunlop et al. (2021) in that the pink salmon subsidies that enter the terrestrial ecosystem provide a benefit for boreal generalist predator/scavenger species. These are species also expected to benefit from ongoing climate change and expected to have negative impacts on endemic arctic biodiversity. The most likely impact of the pink salmon invasion on terrestrial ecosystems in northern Norway is therefore a negative one on already vulnerable arctic species. The white‐tailed eagle is for instance known to have negative impact on sea bird colonies (Anker‐Nilssen et al., 2023) and the red fox to have negative impact on Arctic fox (*Vulpes lagopus*) populations (Elmhagen et al., 2017; Lacombe et al., 2024), a species that is also highly sensitive to climate change (Pedersen, 2021). To what degree the subsidies from pink salmon will affect the population sizes and dynamics of the predator/scavenger community in northern Norway is however, still unknown. The pink salmon become available to the scavenger community when the breeding season of most birds has ended, many migratory bird species have left the study area, and well before the part of the winter season when reindeer carcasses are likely to become available. This may suggest that the pink salmon improve the food availability in a period of the year with limited alternative food resources, and therefore may potentially improve winter survival and subsequent reproduction of the scavenger species. However, the 2‐year spawning cycle of pink salmon is likely to reduce the impact of pink salmon on the population dynamics of scavenger species. Furthermore, the impact of pink salmon on endemic wildlife through effects on scavenger numerical and functional responses is expected to depend on the abundance of non‐migratory alternative prey. In particular, the abundance of small rodents has been shown to influence predator impacts on endemic wildlife (Henden et al., 2021; Marolla et al., 2019; Mellard et al., 2022) and is therefore also expected to affect the consequences of pink salmon on endemic wildlife through the scavenger community.

## AUTHOR CONTRIBUTIONS

**Bror Mathias Bonde:** Conceptualization (lead); data curation (lead); formal analysis (lead); investigation (lead); methodology (lead); project administration (lead); writing – original draft (lead); writing – review and editing (equal). **Audun Stien:** Conceptualization (supporting); data curation (supporting); formal analysis (supporting); methodology (supporting); supervision (lead); writing – original draft (supporting); writing – review and editing (equal).

## CONFLICT OF INTEREST STATEMENT

The authors declare that they have no competing interests.

## Supporting information


Figure S1:

## Data Availability

The dataset and R script used to run the analysis and produce the figures are stored as a dataset in dataverse.no with permanent link: https://doi.org/10.18710/PWUQBP.
