# Supplementary material for: Ecological implications of the pink salmon invasion in northern Norway—Aggregative responses and terrestrial transfer by white‐tailed eagles
Source: Ecol Evol. 2024 Jul 21;14(7):e70001. doi: 10.1002/ece3.70001 (PMC11260996; doi:10.1002/ece3.70001)
Supplement: Supplementary file 1 — Figure S1: [file ECE3-14-e70001-s001.docx]

Supporting information

**
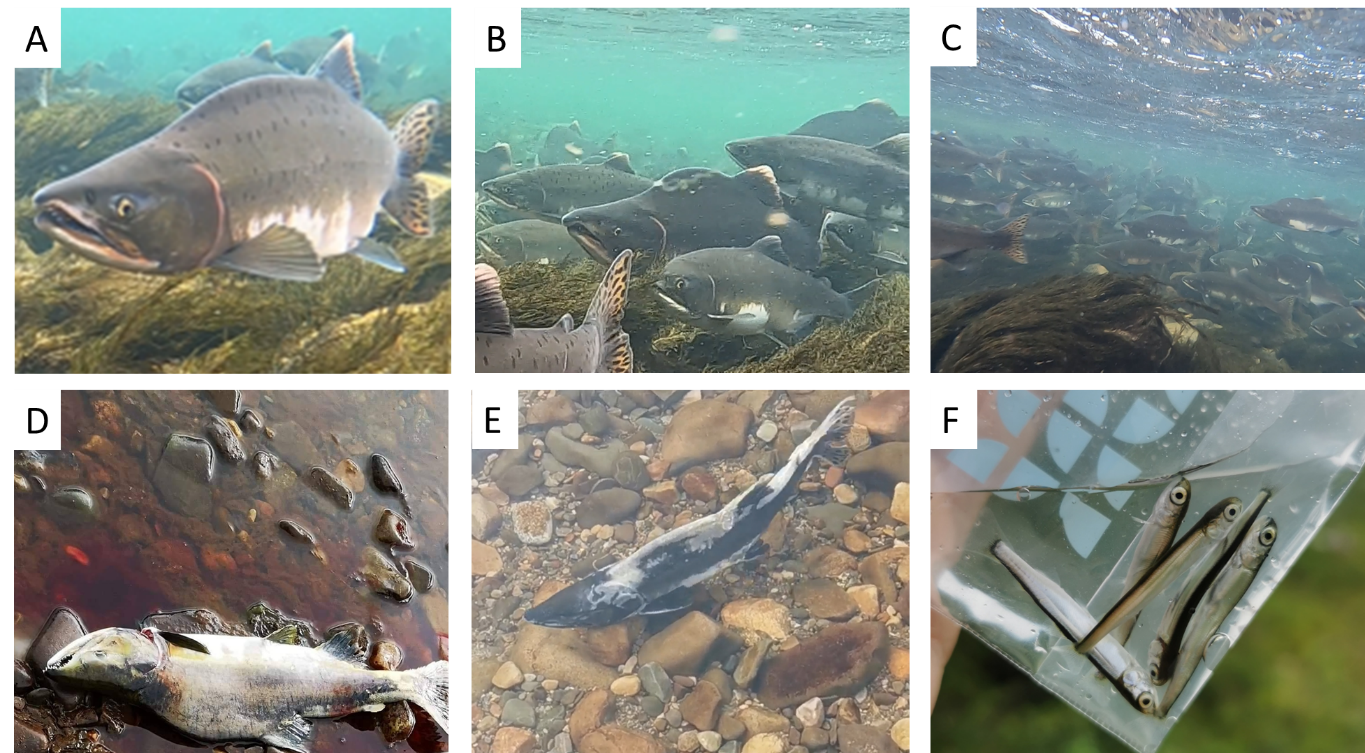
Figure A1. A,** a male pre-spawner that has not yet developed the pronounced hump and curved jaw; **B,** central is a male spawner that had a bleeding bite mark on his large hump and with a pronounced curved jaw with sharp-elongated teeth; **C** illustrates the high density of late pre-spawners encountered on drift counts at Skallelv in July in 2021 making it difficult to count individuals; **D,** a carcass on a mid-channel bank; **E,**  a male post-spawner with open wounds and pronounced deterioration of a large part of its body, including the loss of parts of its tail fin and dorsal fins; **F,** five pink salmon fry caught at Skallelv in July 2022.
